# Theranostics and artificial intelligence: new frontiers in personalized medicine

**DOI:** 10.7150/thno.94788

**Published:** 2024-03-25

**Authors:** Gokce Belge Bilgin, Cem Bilgin, Brian J. Burkett, Jacob J. Orme, Daniel S. Childs, Matthew P. Thorpe, Thorvardur R Halfdanarson, Geoffrey B Johnson, Ayse Tuba Kendi, Oliver Sartor

**Affiliations:** 1 Department of Radiology, Mayo Clinic Rochester, MN, USA.; 2 Department of Oncology, Mayo Clinic Rochester, MN, USA.; 3 Department of Immunology, Mayo Clinic Rochester, MN, USA.; 4 Department of Urology, Mayo Clinic Rochester, MN, USA.

**Keywords:** artificial intelligence, machine learning, theranostics, tumor dosimetry, drug discovery, nuclear medicine

## Abstract

The field of theranostics is rapidly advancing, driven by the goals of enhancing patient care. Recent breakthroughs in artificial intelligence (AI) and its innovative theranostic applications have marked a critical step forward in nuclear medicine, leading to a significant paradigm shift in precision oncology. For instance, AI-assisted tumor characterization, including automated image interpretation, tumor segmentation, feature identification, and prediction of high-risk lesions, improves diagnostic processes, offering a precise and detailed evaluation. With a comprehensive assessment tailored to an individual's unique clinical profile, AI algorithms promise to enhance patient risk classification, thereby benefiting the alignment of patient needs with the most appropriate treatment plans. By uncovering potential factors unseeable to the human eye, such as intrinsic variations in tumor radiosensitivity or molecular profile, AI software has the potential to revolutionize the prediction of response heterogeneity. For accurate and efficient dosimetry calculations, AI technology offers significant advantages by providing customized phantoms and streamlining complex mathematical algorithms, making personalized dosimetry feasible and accessible in busy clinical settings. AI tools have the potential to be leveraged to predict and mitigate treatment-related adverse events, allowing early interventions. Additionally, generative AI can be utilized to find new targets for developing novel radiopharmaceuticals and facilitate drug discovery. However, while there is immense potential and notable interest in the role of AI in theranostics, these technologies do not lack limitations and challenges. There remains still much to be explored and understood. In this study, we investigate the current applications of AI in theranostics and seek to broaden the horizons for future research and innovation.

## Introduction

Theranostics combines advanced imaging techniques with targeted therapy, offering insights into critical areas of precision oncology, such as optimized treatment regimens and efficient monitoring. Although early outcomes from trials and registries have been favorable 1-5, modern theranostics is still in early development. Therefore, many clinical nuances, including individualized dose determination or predictors of treatment success, require further exploration.

Artificial intelligence (AI) comprises advanced computational algorithms designed to recognize patterns in intricate datasets and perform tasks by replicating human intelligence. Given modern data-processing capabilities, AI applications in other sophisticated fields such as radiomics, genomics, or transcriptomics may accelerate advancements in patient management by offering valuable insights into the prediction of outcomes and enriching personalized health care, which is the ultimate goal of theranostics 6.

Despite the incorporation of AI into various levels of healthcare already, its role in theranostics applications deserves further exploration. In this article, we review current and emerging AI applications on important aspects of theranostics, such as patient selection, tumor dosimetry, patient monitoring, and drug discovery to introduce novel viewpoints and inspire healthcare professionals toward future AI-driven research in this field.

## The Concept of AI

Before we explore the applications of AI in theranostics, it is essential to be familiar with some basic terminology. AI is the broadest concept that encompasses all algorithms enabling machines to execute human-like cognitive functions, such as problem-solving, reasoning, and learning 7, 8. Machine learning (ML), deep learning (DL), and neural networks (NNs) are all subsets of AI 9. It is essential to highlight that employing more advance algorithms and more extensive data leads to more accurate outcomes and streamlines the execution of complex tasks. As such, distinctions among these concepts (ML, DL, ANNs) can be made based on nuances such as learning methods and the required volume of data 7.

Machine learning, a comprehensive subset of AI, can learn from 'structured data,' enabling it to identify patterns and learn and perform specific tasks 9. Structured data is well-organized and formatted, usually presented in numbers or letters such as dates or names, making it quickly processed by ML algorithms 7. In contrast, unstructured data, which involves formats like images or texts, lacks a predetermined organization or format, making it more complex and challenging to analyze 10. Although unstructured data offers a more in-depth understanding of context, it requires more sophisticated algorithms than those used for ML.

Neural networks (NNs) are a subset of machine learning (ML), with multiple nodes attempting to simulate human neurons and their interactions 9. These nodes are arranged as one input layer, one or more hidden layers, and one output layer 11. While a single-layer neural network can generate preliminary predictions and/or decisions, incorporating additional layers and more extensive data enhances the quality of outcomes. Neural networks come in various forms, each suited to different types of data and purposes. Convolutional neural networks (CNNs), a specialized type of ANN, employ advanced mathematical operations to analyze and recognize visual data, holding immense potential in the field of radiology 9.

Deep learning represents the most evolved concept within AI, utilizing sophisticated computer programming and extensive training to decipher complex patterns hidden in large datasets 7. Deep learning consists of multiple NNs to execute highly sophisticated tasks with remarkable accuracy. Essentially, the term 'deep' refers to multiple layers of NNs, enabling them to handle complex tasks more effectively 12. Furthermore, unlike traditional ML, which processes structured data, DL extends its capabilities to analyze unstructured data without human intervention. Since unstructured data constitutes a larger portion of currently available data, DL is poised to shape the future of technology.

## AI in Theranostics

Modern molecular imaging technology generates abundant imaging data. These data ultimately require sophisticated automated tools and software systems. Since the introduction of the first FDA-approved AI-enhanced medical device in 1995, AI has been incorporated into multiple facets of healthcare practices, and over 520 AI/ML algorithms have been approved, the majority falling under radiological and oncological applications 13. In **Table 1**, we provide an overview of FDA-approved AI-based radiological software systems in clinical oncology. However, it is important to highlight that none of them are dedicated solely to theranostics. In this chapter, we explore key areas in which AI applications can significantly contribute to and enhance different aspects of theranostics.

### Patient Selection and Risk Stratification

**Figure 1:**
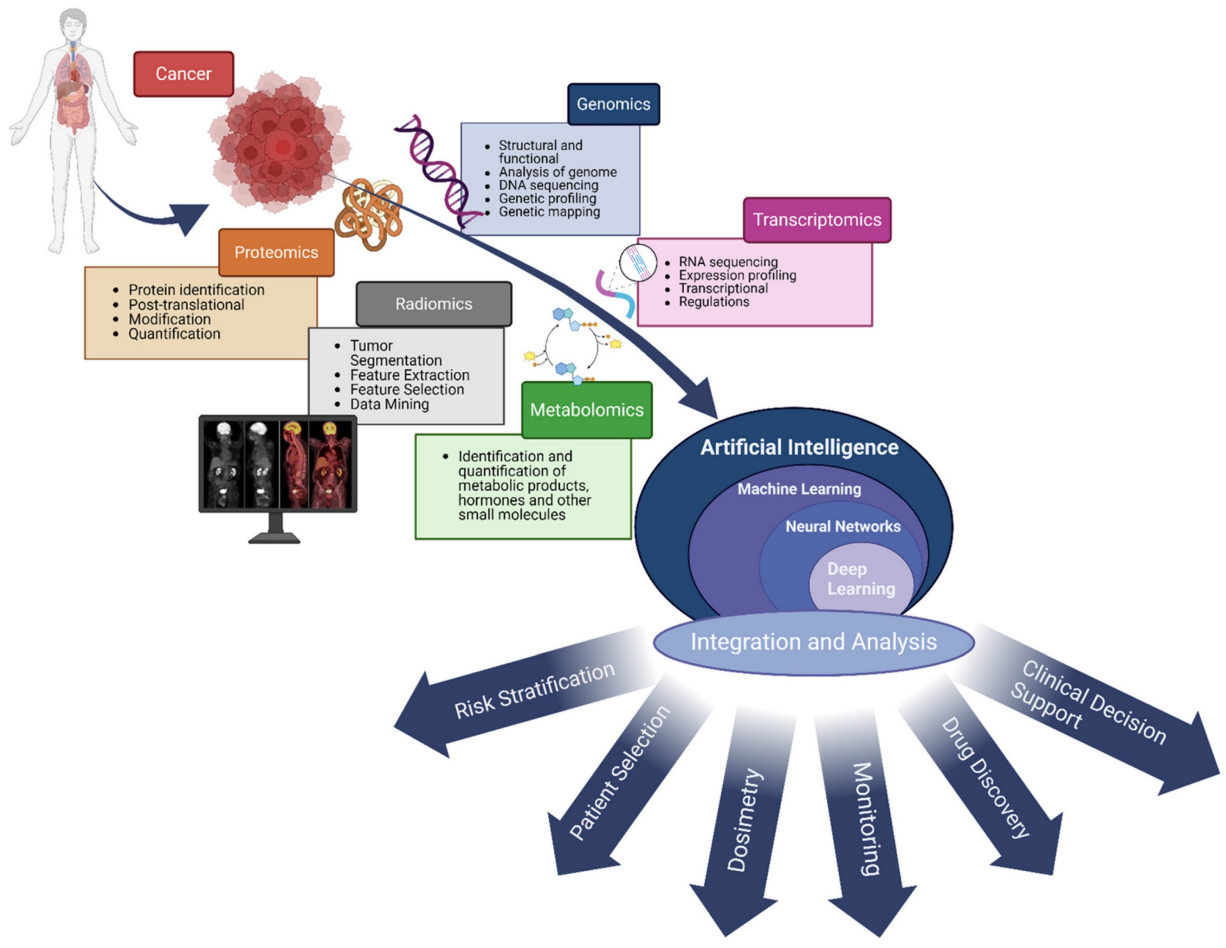
The use of AI to integrate multi-omics biomedical data presents a powerful method for understanding complex biological systems and diseases. Multi-omics data, encompassing genomics, proteomics, metabolomics, transcriptomics, radiomics, and more, offer a comprehensive perspective on the molecular mechanisms that underlie health and disease. AI algorithms, especially those in machine learning and deep learning, excel at analyzing and integrating these heterogeneous datasets, revealing patterns, interactions, and insights that might not be detectable by human eyes. This empowers researchers and clinicians to deepen their understanding of disease pathology, improve patient selection, aid in dosimetry and drug discovery, and develop personalized treatment strategies. Harnessing AI's power in integrating multi-omics data marks a significant leap forward for precision medicine and healthcare advancements.

Given the unique nature of each patient, aligning patients with the optimal treatment options necessitates a thorough analysis taking into account both the patient's individual characteristics and the tumor's specifics. Artificial intelligence holds strong promise for this process by integrating diverse types of patient- and tumor-specific data, including multi-omics (such as genomics and proteomics) **(Figure 1)**. This integration enables a deeper understanding of complex biological systems and diseases, thereby improving the process of selecting the most suitable treatment for each patient.

Traditional cancer stratification mainly relies on the characterization of cancer, including pathological and radiologic features, genetic signatures, and serum markers 14, 15. However, manual interpretation of histopathologic samples and radiologic imaging might be prone to interobserver variability.

Furthermore, observers may not fully capture the intricate diversity of tumor characteristics due to the inherent limitations of human eyes. For instance, current guidelines for using ^177^Lu-PSMA-617 in prostate cancer recommend molecular imaging to evaluate tumor PSMA expression (^68^Ga/^18^F-PSMA PET or ^99m^Tc-PSMA SPECT/scintigraphy) 16. While a significant proportion of patients with high PSMA uptake show a noticeable prostate-specific antigen (PSA) response, 30% still do not achieve satisfactory PSA decline 1. Likewise, ^68^Ga-DOTATATE PET/CT is the preferred method for assessing somatostatin receptor (SSTR2) expression and guiding radionuclide therapy (RNT) candidate selection 17. Notably, even when high ^68^Ga-DOTATATE uptake suggests increased SSTR2 expression in tumor cells, RNT is not effective for all patients 18-20. It is essential to recognize that the efficacy of RNT (Radioligand Therapy) is influenced by a variety of factors, not solely receptor expression. For instance, the radiation absorbed dose by the tumor which is constrained by organs at risk with varying tolerance levels to radiation. Furthermore, as dose-effect relationships still remains uncovered, many tumors may remain underdosed. However, since the current selection criteria for most RNTs are based on receptor expression, the discrepancy in treatment response among those with high receptor levels highlights a gap in understanding the uncovered factors influencing treatment efficacy.

**Figure 2:**
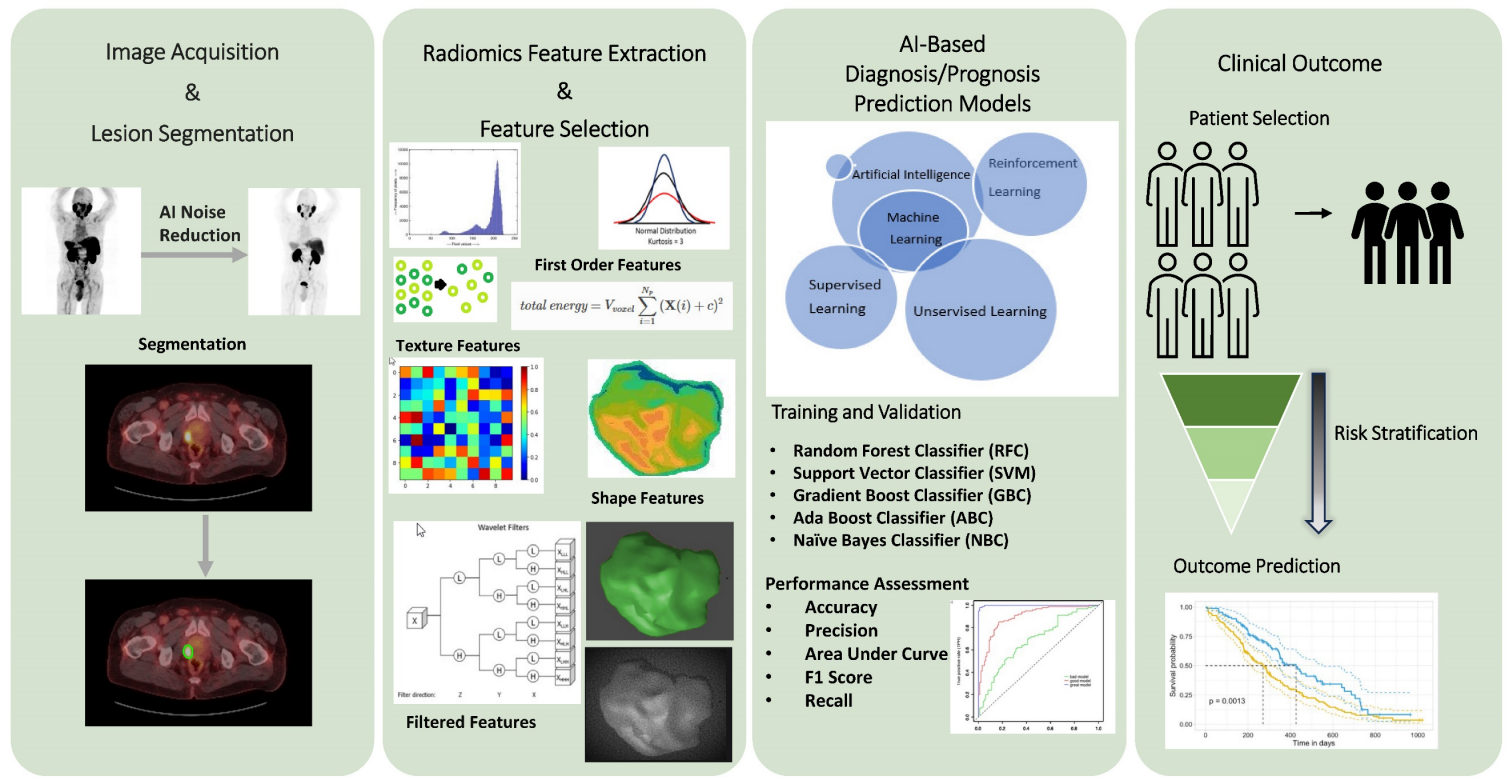
The radiomics workflow incorporates AI-based algorithms to analyze medical imaging data. The radiomics workflow employs AI-based algorithms to extract numerous features from medical imaging data. These algorithms enhance both the patient and physician experience by reducing image acquisition time, aiding in noise reduction, and automating lesion segmentation, without compromising quality. Utilizing radiomic data, AI software transforms images into high-dimensional, mineable data, facilitating the identification of patterns and biomarkers not visible to the human eye. These features can then be correlated with clinical outcomes to enhance diagnostic accuracy, predict disease progression, and personalize treatment plans.

Recently, there has been a growing interest in employing radiomics-based AI models to identify imaging biomarkers for tumor characterization 21-24. Radiomics, fundamentally, is a quantitative method that transforms imaging data into actionable clinical information. Kitajima et al. demonstrated that the imaging biomarker, developed using AI software trained on pre- and post-therapy bone scan images, effectively distinguished the responders and the non-responders of ^223^RaCl_2_ therapy 25. Papp et al. explored the potential of their ML models, trained with PET/MRI radiomic data, to differentiate between low and high-risk prostate lesions and predict biochemical recurrence in patients with prostate cancer 26. They found that radiomic-trained supervised ML models can yield highly efficient noninvasive lesion characterization with 87% sensitivity and 94% specificity. Another study by Bevilacqua et al. assessed the value of radiomic features derived from the hybrid ^68^Ga-DOTANOC PET/CT in determining the histological grading of pancreatic NETs (panNETs) 27. Their models were trained using imaging and histopathologic data from excised primary lesions or biopsies. In their study, the radiomic model predicted histopathologic grade with an 88% sensitivity and an 89% specificity for panNET. These studies demonstrate that AI can predict tumor grade and metastatic potential for certain cancers, such as prostate cancer and NETs 25-28. Therefore, implementation of AI into clinical decision-making processes may yield a result in better risk stratification and patient selection for theranostic applications** (Figure 2)**.

To effectively leverage AI technology, it is required to ensure that the AI models are trained with a broad spectrum of data. This enhances their ability to generalize and apply the knowledge to unseen scenarios. Cysouw et al. established two ML models to assess their predictive capabilities on metastatic disease and high-risk pathological tumor features 28. One model was trained with ^18^F-DCFPyL PET/CT radiomic data, while the other was trained with standard PET features such as SUVs, and volumetric data. The study demonstrated that the radiomic-based models outperformed the model trained with traditional PET parameters, particularly in predicting lymph node involvement and high-risk pathological tumor features. In this study, the predictive power of ML models is significantly enhanced by incorporating broader radiomic data from PET/CT compared to PET-only data, highlighting the importance of comprehensiveness and diversity of training dataset of ML models. Another critical point is AI algorithms continuously evolve and improve over time as it processes more data. However, it is important to provide accurate and standardized imaging data to ensure consistency and reliability of the outcome. This will also help address technical issues affecting the robustness of radiomic features and develop biomarkers for use in diverse clinical settings.

Clinical outcomes of theranostic applications can significantly differ among patients, even those at the same stage and share similar demographics 1, 10, 29. Many factors may play crucial role in determining the response to treatment, such as tumor microenvironment, tumor biology and molecular profile, metastatic burden, and more. Among these potential explanations of response variability intrinsic tumor radiosensitivity also emerges as a critical factor. This radiosensitivity may be influenced by diverse biological and pathological factors such as hypoxia, genetic mutations, and different cell cycle stages 30. In 2022, Kim et al. created an DL model trained on genomic data from various cancer types to detect variations in response to radiation therapy 31. Their DL model predicted in vitro radiosensitivity with an impressive accuracy of 98.85%. Building on this, Dromain et al. explored AI's role in predicting response variability and survival rates in enteropancreatic NETs 32. They defined response heterogeneity as the simultaneous presence of responsive and non-responsive lesions within a single patient. Their DL model, trained on CT images of enteropancreatic NETs, effectively predicted progression-free survival after 12 weeks of treatment. Moreover, the heterogeneity observed between various metastatic lesions has been linked to tumor progression. In light of these results, understanding the response heterogeneity could offer more clinical insight than currently provided by RECIST (Response Evaluation Criteria in Solid Tumors), allowing for predictions of patient outcomes without solely depending on tumor size criteria. These predictions are critical, as decisions to stop or continue treatment at this stage have the potential to reduce the burden of ineffective treatment-related morbidity significantly.

Accurate disease staging and tumor burden quantification are imperative for patient-specific risk classification and evidence-based management. However, the potential for variations in observer assessments can challenge the accuracy of staging 33. Standardizing the staging process can improve diagnostic consistency and sharpen the precision of clinical judgments. Furthermore, precise tumor burden quantification might be a valuable tool for assessing an individual's risk and prospective prognosis. Studies have shown that parameters such as metabolic tumor volume (MTV), an imaging-derived metric, serve as predictors for outcomes in various types of cancer treated with RNT 34-36. While manual evaluations of tumor burden offer crucial insights, they are labor-intensive, which hampers the routine use of volumetric parameters like MTV in clinical practice. The automated platform, PYLARIFY AI™ (aPROMISE), stands out as the only FDA-cleared ML-driven software that provides standardized PSMA PET reporting on PSMA PET/CT images 37, 38. It employs AI to provide quantitative measurements and enhance consistency across various readers. Preliminary findings suggest that aPROMISE could also upstage patients initially evaluated by physicians 38. FDA clearance of such AI-driven technologies signifies a pivotal milestone in theranostics, indicating the potential of AI to augment physician judgment and optimize overall patient management strategies.

### Tumor Dosimetry

Internal radiation dosimetry quantifies the amount of ionizing radiation energy deposited per unit mass or voxel of organ/tissue 39. By predicting and identifying the absorbed radiation doses by the target, organ-at-risk, and healthy tissues, dosimetry studies provide essential insights into the objective assessment of a treatment's safety and efficacy for each patient 40. Nonetheless, internal dosimetry is rarely performed in nuclear medicine clinical settings, unlike its widespread application in radiotherapy clinics. This difference is primarily because of the complex dynamics of radionuclides within the body and the time-intensive processes associated with traditional dosimetry techniques. In a routine practice, the dosage of radioactivity in theranostics is administered either as a fixed activity dose or adjusted based on the patient's weight. However, it is important to note that the absorbed dose is influenced by various well-known internal and external factors, such as the physical half-life, type of emitted radiation, biological distribution and elimination rates, tissue weighting factors, patient and tumor characteristics, highlighting the importance of adopting personalized dosimetry approaches.

Given that only trace amounts of radioactivity are used for diagnostic studies, the risks of radiation-induced damage to healthy tissue are often considered negligible 41. However, for therapeutic purposes, radionuclides are also commonly administered in standardized dosages, like the administration of diagnostic purposes. Even though these doses are established to ensure a therapeutic success while minimizing toxicity risks, a one-size-fit-all approach may overlook individual variabilities, particularly at substantially higher doses. In addition, despite encouraging progression-free and overall survival results of radionuclide therapies, some patients may need retreatment, which increases cumulative radiation exposure and likelihood of radiation-induced damage 42. Therefore, adopting standardized approach could result in either undertreatment or overtreatment of the disease, or exceed safe radiation dose thresholds for organs-at-risk 43, 44.

One unique aspect of theranostics is its capability to gather pharmacokinetic data through imaging and apply this information to dosimetry calculations 45. To acquire the imaging data, various imaging techniques like planar scintigraphy, SPECT/CT, or PET/CT can be utilized for tracking the radionuclide before or after therapy. The application of these imaging modalities at different stages—pre-therapy and post-therapy—yields unique insights. While pre-therapy imaging is pivotal for individualized treatment planning, post-therapy imaging provides critical data on the treatment's outcome, including the distribution and elimination of radionuclides, offering insights into therapy's impact on the target and surrounding tissues 46, 47. However, in a busy clinical setting, acquiring multiple pre/post-therapy images at various times within a specific timeframe is often considered impractical for both healthcare providers and patients. Therefore, the demand for feasible dosimetry methods has increasingly grown, particularly following the widely availability of diverse radionuclide therapies 48, 49. Among these, the Hänscheid and prior-information approaches stand out as the methods to simplify dosimetry in theranostics. The Hänscheid approach employs a single SPECT/CT scan at a specific time after each cycle of radionuclide therapy to calculate absorbed doses 50. The prior-information approach uses time-activity curve models from multiple SPECT/CTs after the initial cycle, applying these models to single-time-point acquisitions in subsequent therapy cycles 51. In light of the growing body of research supporting the effectiveness of time-efficient dosimetry methods 47-49, 52, the integration of AI technologies, specifically CNN and DL, holds significant promise for further optimizing and enhancing these processes. Training CNN and DL models with extensive datasets from prior dosimetry studies and pre/post-therapy images enables the identification of patterns that correlate physiological parameters with the kinetics of radionuclides.

Well-established dosimetry methods like the Medical Internal Radiation Dose (MIRD) system combine biological distribution and clearance data and is considered ideal for organ-based dosimetry 53. However, traditional MIRD methods relied on a simplified, non-specific model of a 70 kg adult male or female as a phantom for these dosimetry calculations, resulting in a lack of patient-specific details 54. Another prime example of dosimetry techniques is Monte Carlo (MC) simulations 55. Monte Carlo simulations provide in-depth predictions of radiation dose distributions within the human body, taking into account complex bodily structures and radiation-tissue dynamics 56. Despite the advantages of MC simulations dosimetry, its high computational demands to perform complex equations and algorithms limit its clinical use 45. To address the limitations and harness the strengths of traditional dosimetry techniques, an increasing number of studies are utilizing them to train DL models. Several studies have demonstrated that absorbed dose estimation using DL models is on par with or even exceeds traditional methods 52, 57-60. Thus, by combining established methodologies with cutting-edge DL tools, manual labor and time might be significantly reduced while enhancing accuracy.

AI aids in both pre- and post-treatment dosimetry by facilitating organ and tumor segmentation, reducing errors and segmentation time. For instance, Sharma et al. reported experts require approximately 30 minutes to segment a kidney 61; in contrast, Nazari et al. demonstrated an AI-driven system segmenting both kidneys in less than 3 seconds with high accuracy 62. However, these systems are not flawless. AI tools designed for specific cancer types may not be universally applicable. Indeed, this "Clever Hans Effect"—a phenomenon where an AI tool excels at a particular task but struggles to generalize to different scenarios—is prevalent in AI-driven dosimetry 63, 64. For example, the PET-Assisted Reporting System prototype uses NNs trained on lymphoma and lung cancer data. However, its accuracy in interpreting ^18^F-FDG PET/CT scans for other tumors remains uncertain 64.

Researchers have also explored various strategies to accelerate image acquisition time 65. One approach involves reducing the number of projections or the time spent per projection. However, brief acquisition times can lead to heightened image noise, potentially impacting image quality and tumor-to-background ratio. This challenge can be addressed by using AI-based learnable systems, like image-reconstruction neural networks. For instance, Shao et al. developed a DL-model, called SPECTnet, that directly converts raw projection data into high-resolution, low-noise images 66. By employing a novel two-step training strategy and utilizing a vast dataset of 2D phantoms, the research demonstrates the capability of their DL-model to produce more clear SPECT images compared to traditional reconstruction methods in less than a second.

The utilization of AI-based tools in dosimetry studies for theranostics holds the promise of significantly enhancing study accuracy and efficiency. However, further research is still needed to validate their effectiveness and integrate them into routine clinical practice.

### Disease Monitoring

Numerous studies have demonstrated that semi-quantitative PET parameters and their variations throughout treatment provide valuable insights into disease prognosis and the effectiveness of the treatment 67, 68. For instance, in prostate cancer, changes in tumoral volume are considered a biomarker for defining response to RNT 69. By harnessing adaptive learning algorithms, AI holds the revolutionary potential to leverage traditional parameters to advance therapy monitoring. Various AI-based platforms offer e-consults to clinicians and patients across numerous specialties, enriching and refining the clinical experience. For instance, Tempus Labs, an AI-enabled clinical assistant, identifies genetic mutations in tumor tissue samples, analyzes electronic health records, lab values and imaging data, and combines all patient-specific information to create a comprehensive medical profile. Utilizing this profile, Tempus Labs provides healthcare professionals with personalized therapy recommendations in real-time via interactive systems to facilitate the decision-making process throughout the treatment course.

Leveraging electronic health record data and variations of the lab data, AI-driven algorithms may contribute to optimized patient care for cancer patients. These algorithms assess and triage patients, enabling healthcare professionals to tailor follow-up appointments and interventions based on individual needs and acuity 70. This tailored approach aims to ensure judicious resource management and augment the patient's healthcare experience, potentially leading to lower mortality and morbidity rates.

**Figure 3:**
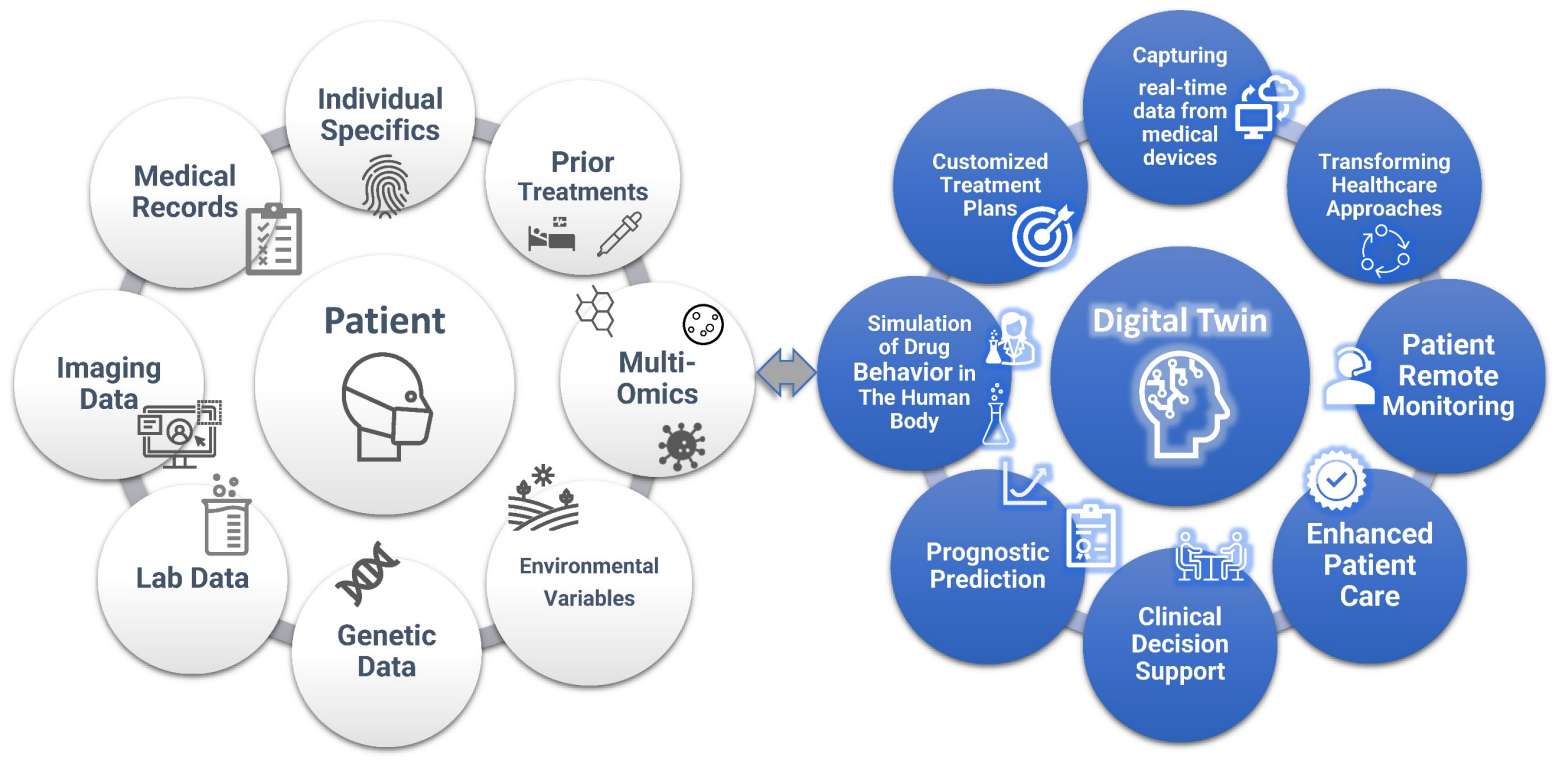
The concept of “Digital Twin”. Digital twins in healthcare are virtual models that replicate an individual's health status, integrating data from a wider variety of sources such as medical records, imaging data, lab tests, genetic data, environmental variables, prior treatments, and multi-omics. These models enable personalized treatment planning, predictive analytics for disease progression, and the optimization of healthcare delivery. By simulating different medical scenarios and capturing real-time data from medical devices, digital twins can improve patient outcomes through tailored interventions and proactive health management. In addition, digital twins support clinical decision process and transform healthcare.

As a future direction, a digital twin is a dynamic virtual model that mimics the behavior of a real object or system, enhanced by real-time data, simulation, and ML. While this is a relatively novel concept in healthcare, NASA has been using a similar concept since the 1960s by creating Earth-based simulations of spacecraft for study and simulation. In the medical realm, digital twins represent virtual replicas of patients, aiding clinicians in decision-making 71, 72. The models incorporate a wide range of data, such as environmental variables, medical records, medical imaging, laboratory data, individual characteristics, genetic information, and prior treatment histories **(Figure 3)**. Also, unlike traditional simulations or models, digital twins learn and update over time. This allows them to adapt to changing circumstances in real-time, just as humans do 73. Thus, digital twins offer a promising approach to creating customized treatment plans, taking into account factors such as comorbidities or potential drug interactions and providing valuable insight into disease trajectories.

### Drug Discovery

While theranostics have existed for decades, recent advances in the field have stimulated more pharmaceutical companies to develop new radiopharmaceuticals, repurpose existing drugs, and broaden therapeutic applications. Unsurprisingly, the global radiopharmaceutical market is on an upward trajectory, with forecasts predicting its worth to reach 9.53 billion US dollars by 2031 74.

**Figure 4:**
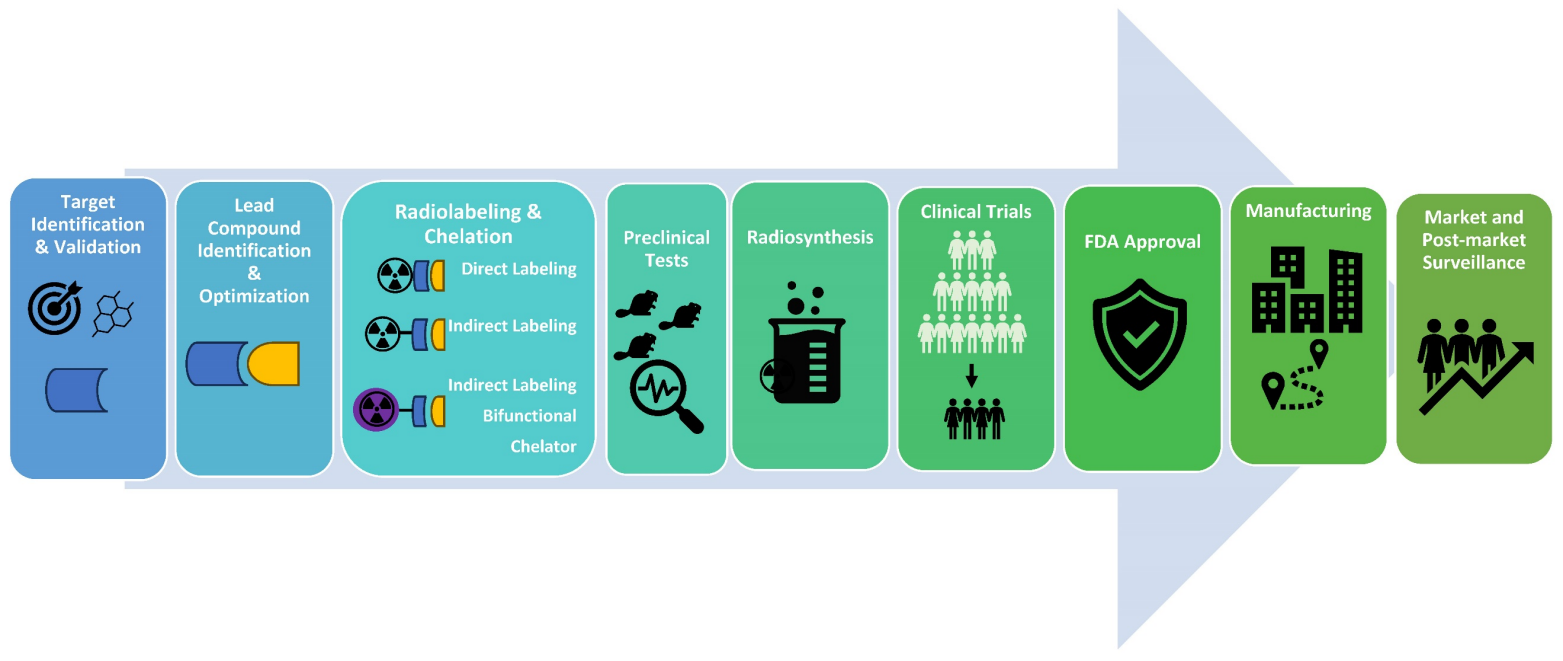
Radiopharmaceutical Drug Development Process. The radiopharmaceutical drug development process involves identifying target molecules and suitable lead compounds, followed by the design, synthesis, and validation of radioactive compounds used for diagnosing or treating diseases. Initially, a potential therapeutic or diagnostic agent is identified and chemically bonded to a radioactive isotope. This compound undergoes rigorous preclinical testing to assess its safety, biodistribution, and efficacy in biological models. Successful candidates advance to clinical trials, where their therapeutic effectiveness, dosimetry, and safety are evaluated in patients. This meticulous process ensures that radiopharmaceuticals are effective for their intended use and safe for patient application. After receiving FDA (US Food and Drug Administration) approval, the process continues with manufacturing and post-market surveillance.

Radiopharmaceutical development is a challenging endeavor, encompassing a myriad of facets including target identification, lead compound identification, radionuclide selection, vector molecule formulation, synthesis, evaluations, and drug approvals **(Figure 4)**. These processes are tedious and costly, but AI-powered theranostics drug discovery studies promise optimization, paving the way for quicker development and approval of innovative radiopharmaceuticals. Therefore, tech behemoths like Google, DeepMind, Insilico Medicine, Deep Genomics, and Healx are also making considerable investments in AI-based drug discovery applications.

The development of drugs is parallel to the discovery of new targets. Incorporating AI can effectively streamline this critical step by automation techniques. For example, the AI tool AlphaFold has been used to analyze the amino acid sequences and the angles of peptide bonds to predict the 3D structure of proteins. Recently, Ren et al. conducted a study demonstrating the application of AlphaFold in identifying potential drug targets 75. Their initial findings revealed a novel CDK20 small molecule inhibitor that may represent a promising avenue for treating hepatocellular carcinoma. In January 2023, Absci, a generative AI drug development company, announced that its AI platform had successfully produced de novo antibodies using innovative AI methodology and validated them in wet lab experiments 76. The unique aspect of their approach is that it is capable of creating antibodies without using training data, thereby reducing both the time and resources typically necessary in the drug discovery process. These recent innovations hold immense promise in the theranostic landscape, where targets range from antibodies to enzymes critical in carcinogenesis.

After identifying a target, subsequent steps assess the identified molecules for their potential as drug candidates. In silico models, computer-based experimentation, and virtual screening (VS) techniques can expedite this assessment using AI algorithms. VS is a computational method that efficiently sifts through vast databases to identify compounds with a high likelihood of desired biological activity against a specified target while simultaneously eliminating potentially harmful or suboptimal candidates. Within the VS process, the composition and structure of identified compounds can be adjusted to optimize pharmacokinetic characteristics such as absorption, distribution, metabolism, excretion, and toxicity 77, 78.

Once ligands have been refined, they are ready for biological evaluation and prediction of an optimal radiolabeling process. This involves selecting the most appropriate method to attach a radioactive isotope to the target molecule, ensuring both efficiency and stability. In addition to its role in theoretical drug design, AI is also employed to generate synthesis pathways for hypothetical drug compounds. In certain cases, AI may also suggest modifications to these compounds that would facilitate their manufacturing process 79.

### AI Limitations

While AI has streamlined many processes in our daily lives, its application in healthcare still necessitates further considerations. Since the effectiveness of AI algorithms relies on the diversity and scope of its training data, limiting its application to rare conditions or under-represented minorities without specific data 63, 64, 80. Also, concerns regarding the privacy and security of patient information remain paramount as AI learns from broad data collections, which may be subject to unauthorized access and misuse 80. Another concern is that AI systems cannot be held accountable for the outcome of their applications. Accountability in AI systems largely depends on the transparency of the algorithms, the quality of training data, and the decisions made by developers and/or users. This highlights the importance of structured guidelines and regulations to ensure the responsible use of AI systems. Despite these hurdles, the integration of AI in healthcare is rapidly expanding every day. Therefore, addressing the current challenges by developing sophisticated, trustworthy AI algorithms should be a focus of interest, aiming to harness AI's full potential responsibly and effectively in healthcare settings.

## Conclusion

AI in theranostics has the potential to revolutionize healthcare by providing personalized, data-driven insights to support clinicians and patients. From tumor characterization, personalized patient risk classification, prognostic forecasts, personalized dosimetry to uncovering potential factors unseeable to the human eye, such as intrinsic variations in tumor radiosensitivity or molecular profile, AI software has the potential to revolutionize the personalized medicine. However, despite these advances holding significant promise, adopting AI algorithms to the routine clinical practice raises several concerns, including security of data, accountability of outcome, and comprehensiveness and diversity of outcomes. Therefore further research and guidelines outlining the responsible use of AI are needed to harness the full potential of AI, while considering its limitations and ensuring its successful integration into clinical practice.

## Figures and Tables

**Table 1:** Overview of FDA-approved AI-based radiological software systems in clinical oncology

Device	Company	Short description	FDA approval number	Date of approval*
***Breast cancer***				
BU-CAD	TaiHao Medical Inc.	Detection of suspicious lesions for breast cancer	K210670	2021
Koios DS	Koios Medical, Inc.	Detection of suspicious lesions for breast and thyroid cancer	K212616	2021
MammoScreen 2.0	Therapixel	Detection of suspicious lesions for breast cancer	K211541	2021
Lunit INSIGHT MMG	Lunit Inc.	Detection of suspicious lesions for breast cancer	K211678	2021
Saige-Q	DeepHealth, Inc.	Detection of suspicious lesions for breast cancer	K203517	2021
Visage Breast Density	Visage Imaging GmbH	The BI-RADS breast density classification	K201411	2021
Imagio Breast Imaging System	Seno Medical Instruments, Inc.	The BI-RADS breast density classification	P200003	2021
PowerLook Density Assessment Software V4.0	ICAD Inc.	The BI-RADS breast density classification	K211506	2021
Volpara Imaging Software	Volpara Health Technologies Limited	The BI-RADS breast density classification	K211279	2021
Genius AI Detection	Hologic, Inc.	Identification of suspicious breast lesions	K201019	2020
Genius AI Detection	Hologic, Inc.	Detection of suspicious lesions for breast cancer	K201019	2020
WRDensity by Whiterabbit.ai	Whiterabbit.ai Inc.	Software for BI-RADS breast density classification	K202013	2020
MammoScreen	Therapixel	Radiological software for lesions suspicious for breast cancer	K192854	2020
HealthMammo	Zebra Medical Vision Ltd.	Identification of suspicious breast lesions	K200905	2020
Densitas Densityai	Densitas, Inc.	The BI-RADS breast density category using mammography	K192973	2020
TransparaTM	Screenpoint Medical B.V.	Identification of suspicious breast lesions	K192287	2019
ProFound AI Software V2.1	ICAD Inc.	Identification of suspicious breast lesions	K191994	2019
cmTriage	CureMetrix, Inc.	Identification of suspicious breast lesions	K183285	2019
TransparaTM	Screenpoint Medical B.V.	Identification of suspicious breast lesions	K192287	2019
Koios DS for Breast	Koios Medical, Inc	Identification of suspicious breast lesions	K190442	2019
PowerLook Tomo Detection V2 Software	ICAD Inc.	Identification of suspicious breast lesions	K182373	2018
ProFound™ AI Software V2.1	iCAD, Inc	The BI-RADS breast density classification using mammography	K191994	2018
DM-Density	Densitas, Inc.	The BI-RADS breast density classification	K170540	2018
Volpara Imaging Software	Volpara Health Technologies Limited	The BI-RADS breast density classification	K182310	2018
DenSeeMammo	STATLIFE	The BI-RADS breast density classification	K173574	2018
***Prostate cancer***				
ProstatID	ScanMed, LLC	The detection and diagnosis of prostate cancer utilizing MRI images	K212783	2022
aPROMISE	EXINI Diagnostics AB	Identification and quantitative analysis of suspicious regions on PSMA PET/CT	K211655	2021
QUIBIM Precision Prostate (qp-Prostate)	QUIBIM S.L.	Software to detect prostate cancer and prostate diseases	K203582	2021
PROView	GE Medical Systems SCS	The PI-RADS prostate density classification using mpMRI	K193306	2020
A View LCS	Coreline Soft Co., Ltd.	The PI-RADS prostate density classification using mpMRI	K201710	2020
Quantib Prostate	Quantib BV	The PI-RADS prostate density classification using mpMRI	K202501	2020
ClearRead CT	Riverain Technologies, LLC	The PI-RADS prostate density classification using mpMRI	K161201	2016
***Lung cancer***				
syngo.CT Lung CAD (Version VD20)	Siemens Healthcare GmbH	Image processing of CT scans for solid-subsolid nodules	K203258	2021
Optellum Virtual Nodule Clinic, Optellum Software, Optellum Platform	Optellum Ltd	Identify of suspected pulmonary nodules	K202300	2021
Auto Lung Nodule Detection	Samsung Electronics Co., Ltd.	Detection of suspected pulmonary nodules	K201560	2021
InferRead Lung CT.AI	Beijing Infervision Technology Co., Ltd.	Identify of suspected pulmonary nodules	K192880	2020
AVIEW LCS	Coreline Soft Co., Ltd	Identify of suspected pulmonary nodules	K193220	2020
Syngo.CT Lung CAD	Siemens Medical Solutions, Inc	Identify of suspected pulmonary nodules	K193216	2020
Arterys MICA	Arterys Inc	Diagnostic imaging for liver, lung cancer	K182034	2018
***Brain***				
NeuroQuant	CorTechs Labs, Inc	Interpretation of MRI brain images	K170981	2017
Quantib Brain 1.2	Quantib BV	Interpretation of MRI brain images	K163013	2017
CT CoPilot	ZepMed	Identification and segmentation of brain structures	K161322	2016
cNeuro cMRI	Combinostics Oy	Identification and segmentation of brain structures	K171328	2018
***Miscellaneous***				
aPROMISE X	EXINI Diagnostics AB	Image processing, quantification and reporting of PET scans	K220590	2022
Koios DS	Koios Medical, Inc.	Detection of suspicious lesions for breast and thyroid cancer	K212616	2021
Saige-Dx	DeepHealth, Inc.	Radiological software for lesions suspicious for cancer	K220105	2022
Discovery MI Gen2	GE Medical Systems, LLC.	PET/CT system for producing attenuation corrected images	K211846	2021
Ezra Plexo Software	Ezra AI Inc.	Software for detecting cancerous lesions in MR images	K192969	2020
QuantX	Quantitative Insights, Inc.	Radiological software for lesions suspicious for cancer	DEN170022	2020
Deep Learning Image Reconstruction	GE Medical Systems, LLC.	CT image reconstruction	K183202	2019
SubtlePET	Subtle Medical, Inc.	Noise reduction algorithm	K182336	2018
Quantitative Total Extensible Imaging (QTxi)	AIQ Solutions, Inc.	Create ROI contours for quantitative/statistical analysis and dosimetry	K173444	2018
Arterys Oncology Dl	Arterys Inc.	Asist with analysis of a suspicious lesion on CT or MRI	K173542	2018
Radiomics App V1.0	Microsoft Corp.	Analysis of CT and MRI for dosimetry purposes	K173420	2017
PixelShine	AlgoMedica	CT noise reduction algorithm	K161625	2016

* Last FDA update on approved AI-enabled medical technologies was released on October 5, 2022. AI: artificial intelligence; CT: computed tomography; FDA: Food and Drug Administration; MRI: magnetic resonance imaging; mpMRI: multi-parametric MRI; ROI: region of interest.
